# Rebooting immunity in congenital athymia: Factors impacting reconstitution with thymus implantation

**DOI:** 10.70962/jhi.20260016

**Published:** 2026-05-26

**Authors:** Samantha Cresoe-Ortiz, Guglielmo Venturi, Elizabeth A. McCarthy, Benjamin Stewart-Bates, Jennifer R. Heimall, Eveline Y. Wu, John W. Sleasman, Geoffrey Hall

**Affiliations:** 1 Division of Allergy and Immunology, Department of Pediatrics, Duke University School of Medicine, Durham, NC, USA; 2 Division of Allergy and Immunology, Children’s Hospital of Philadelphia, Philadelphia, PA, USA; 3 Divisions of Pediatric Rheumatology and Pediatric Allergy/Immunology, Department of Pediatrics, University of North Carolina at Chapel Hill, Chapel Hill, NC, USA

## Abstract

Allogeneic processed thymus tissue-agdc (RETHYMIC) is the only Food and Drug Administration–approved therapy for immune reconstitution in children with congenital athymia. Evaluating factors impacting successful immune reconstitution is necessary to improve outcomes. Using data from 10 open-label, single-arm studies, this retrospective cohort study evaluated 76 children with 1-year survival following thymus tissue implantation. Variables included receipt of immune-suppressing agents, human leukocyte antigen (HLA) matching, preimplantation T cell function, and age at implantation. Outcomes include lymphocyte subsets, T cell function, and naive T cell reconstitution, defined as >100 naive T cells/mm^3^ at 1-year after implantation. Median total T, B, and NK cell counts at 1 year following implantation were not associated with T cell function at implantation, immune-suppressing therapies, or donor and recipient HLA matching; however, naive T cell counts were higher among those with low T cell function at time of implantation. Younger age at implantation was associated with improved immune reconstitution, supporting the importance of early diagnosis and referral for treatment.

## Teaser

Allogeneic processed thymus tissue-agdc (RETHYMIC) is approved therapy for treatment of congenital athymia. Study outcomes include changes in T lymphocyte subsets and T cell function. Immune reconstitution was not associated with T cell function, immune suppression, or HLA matching. Younger age at implantation was associated with improved immune reconstitution.

## Introduction

Congenital athymia (CA) is a rare, life-threatening disorder characterized by the absence of a functioning thymus at birth, most commonly the result of defective pharyngeal pouch development (1, 2). Without a thymus, children are unable to develop functional T cells or establish self-tolerance, thereby predisposing them to life-threatening infections, autoimmune disease, and ultimately death, typically within the first few years of life (3, 4, 5, 6). Multiple molecular and embryonic defects are associated with CA, including 22q11.2 deletion syndrome (22q11.2DS), CHARGE (coloboma, heart defect, choanal atresia, growth or mental retardation, genital hypoplasia, and ear anomalies or deafness) syndrome due mutations in chromodomain helicase DNA-binding protein 7 (*CHD7*), and mutations in T-box transcription factor 2 (*TBX2*), paired box protein Pax-1 (*PAX1*), and forkhead box protein N1 (*FOXN1*) (1, 7, 8, 9, 10). Other causes include maternal diabetic embryopathy and intrauterine exposure to isotretinoin (5). These conditions disrupt pharyngeal pouch development during early embryogenesis, resulting in midline syndromic anomalies affecting the heart, parathyroid gland, facial structures, and thymus (5, 11). All newborns with CA lack recent thymus emigrants that can be detected with newborn screening for congenital T cell deficiencies (12, 13). The diagnosis is based on low naive T cell numbers at birth (<50 cells/mm^3^ or <5% of total CD3 T cells), presence of syndromic features associated with CA, and the absence of evidence for other T cell immune deficiencies such as severe combined immunodeficiency (SCID) (5, 12, 14).

In the United States, allogeneic processed thymus tissue-agdc (RETHYMIC) is the only definitive therapy approved by the Food and Drug Administration for treatment of pediatric patients with CA (15). Approval of the Biologics License Application occurred in October 2021 and was based on a series of Investigational New Drug (IND) studies that included 105 pediatric patients with CA treated at Duke University in Durham, NC (16). These children received cultured thymus tissue implantation (CTTI) between 1993 and 2020 (15). The majority who received CTTI were included in the efficacy outcome cohort analysis with the primary outcome as T cell reconstitution after 1 year (16). The kinetics of T cell reconstitution following CTTI varied across cohorts with few children displaying detectable naive T cells before 9 mo after implantation. Prior to CTTI, nearly half (45%) of recipients showed signs of aberrant T cell development defined as detectable memory T cells in the peripheral blood and evidence of proliferative responses to mitogens (3). Many of these children had signs of autoimmunity and required immune suppression prior to CTTI. Further complicating the success of CTTI, infants with functional T cells prior to CTTI are capable of allorecognition and cellular rejection of the thymus implant (17, 18). Taken together, immune suppression is a key component in the management of CA along with receipt of CTTI. To date, there has not been a detailed analysis of variables associated with T cell reconstitution among the children with CA included in the efficacy outcome cohort, including the degree of HLA mismatch between thymus donor and recipient, pre-CTTI T cell function (measured by proliferation response to phytohemagglutinin [PHA] mitogen), the use of immune suppression, or age of recipient at the time of implantation. The purpose of this study is to elucidate how these factors affect immune reconstitution following CTTI in children with CA who were enrolled in the initial clinical trials and survived for at least 1-year after treatment.

## Results

### Demographic characteristics of participants in the efficacy outcome cohort

97 participants were included in the efficacy outcome cohort and treated with CTTI under IND 9836 (16). The demographics, syndromic features, and comorbidities within this cohort are shown in Table 1. Greater than 90% of the cohort displayed cardiac, facial, parathyroid, or renal abnormalities associated with abnormal pharyngeal pouch development. Many had evidence of autoimmunity, including autoimmune rash due to autoreactive T cell infiltrates confirmed by biopsy, autoimmune cytopenias, and autoimmune thyroid disorders. Two participants had symptomatic graft-versus-host disease (GVHD), one associated with maternal T cell engraftment and the other associated with a nonirradiated blood transfusion (16).

**Table 1.** Demographic characteristics of efficacy outcome cohort

​	Cohort (*n* = 97)
**Sex, *n* (%)**	​
Male	58 (60)
**Race, *n* (%)**	​
White	68 (70)
Black, African American	21 (22)
Asian	3 (3)
American Indian	2 (2)
Native Hawaiian	1 (1)
>1 race	2 (2)
**Ethnicity, *n* (%)**	​
Hispanic or Latino	18 (19)
Not Hispanic or Latino	79 (81)
**Genetic/syndromic etiology, *n* (%)**	​
22q11.2 deletion	36 (37)
CHARGE/*CHD7* mutation^a^	24 (25)
*FOXN1* mutation	2 (2)
*PAX1* mutation	1 (1)
*TBX2* mutation	1 (1)
Diabetic embryopathy	21 (22)
No known mutation/syndrome	12 (12)
**Syndromic comorbidities, *n* (%)**	​
Choanal atresia	11 (11)
Cleft palate or submucous cleft	17 (17)
Coloboma	19 (20)
Congenital cardiac anomaly	87 (90)
Deafness or ear pinnae anomalies	50 (51)
Dysmorphic facies	45 (46)
Tracheal anomalies	25 (26)
Genital hypoplasia	12 (12)
Hypocalcemia	82 (84)
Anal/rectal anomalies	6 (6)
Renal anomalies	26 (27)
**Autoimmunity, *n* (%)**	​
Rash^b^	37 (38)
Symptomatic GVHD^c^	2 (2)
Thrombocytopenia	10 (10)
Neutropenia	6 (6)
Autoimmune hemolytic anemia	6 (6)
Hypothyroid	6 (6)
**Age at time of implantation (months)**	​
Median (Q1; Q3)	9 (4; 14)
Minimum, maximum	1, 53

a CHARGE/*CHD7* numbers are based on phenotype or genetic testing.

b Based on skin biopsy.

c Due to maternal T cells or nonirradiated blood transfusion.

### Demographic characteristics of study cohort

**Figure 1.**
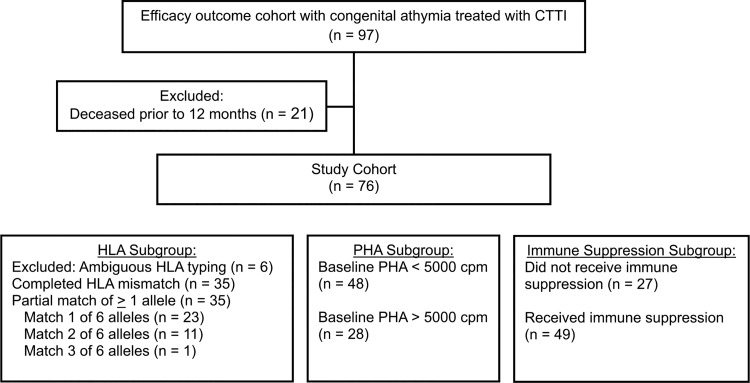
Study design flowchart.

This study includes analysis of a subset of the original 97-participant efficacy outcome cohort, excluding the 21 participants (21.6%) who died prior to reaching 12 mo after CTTI (Fig. 1). Baseline demographics of the remaining 76 participants are displayed in Table 2 showing 54% male, 72% white, and 82% non-Hispanic. Genetic defects associated with CA included 22q11.2DS in 34% (*n* = 26), CHARGE syndrome in 28% (*n* = 21), *FOXN1* mutation in 3% (*n* = 2), *PAX1* mutation in 1% (*n* = 1), *TBX2* mutation in 1% (*n* = 1), diabetic embryopathy in 22% (*n* = 17), and no identified cause for CA in 11% (*n* = 8). Across the cohort, the median (Q1; Q3) age at time of CTTI was 8.9 mo (4.6; 14.1 mo). The 76 participants included in the analysis were categorized into three analysis subgroups (Fig. 1). The HLA subgroup was based on HLA typing between thymus donor and recipient. Within the 76 participants in this subgroup, 35 were complete mismatches at all six HLA alleles, and 35 had partial match for at least one HLA allele. Six participants had ambiguous (incomplete) HLA typing and were excluded from the analysis. The PHA subgroup was based on proliferative responses to PHA mitogen prior to CTTI. 48 participants had PHA responses <5,000 count per minute (cpm), and 28 participants had PHA responses >5,000 cpm. The immune suppression subgroup was based on a history of having received immune suppression therapy preceding, during, or following CTTI. This subgroup included 49 participants who received immune suppression and 27 who did not. Within the 76 participants, three (4%) received alemtuzumab for treatment autoimmune comorbidities, one at 3 mo and two at 13 mo after CTTI, which results in T cell depletion. Their results were censored from subsequent analysis. Outcomes after 25 mo for the participants who received alemtuzumab are shown in Table S1.

**Table 2.** Demographic characteristics of study cohort

​	Study participants (*n* = 76)
**Sex, *n* (%)**	​
Male	41 (54)
**Race, *n* (%)**	​
White	55 (72)
Black, African American	16 (21)
Asian	3 (4)
American Indian	1 (1)
Native Hawaiian	1 (1)
**Ethnicity, *n* (%)**	​
Hispanic or Latino	14 (18)
Not Hispanic or Latino	62 (82)
**Genetic/syndromic etiology, *n* (%)**	​
22q11.2 deletion	26 (34)
CHARGE/*CHD7* mutation^a^	21 (28)
*FOXN1* mutation	2 (3)
*PAX1* mutation	1 (1)
*TBX2* mutation	1 (1)
Diabetic embryopathy	17 (22)
No known mutation/syndrome	8 (11)
**Age at time of implantation (months)**	​
Median (Q1; Q3)	8.9 (4.6; 14.1)
Minimum, maximum	1.1, 53.1
**HLA matching**	​
Complete mismatch (6/6)	35 (46)
Partial match (>1)	35 (46)
Ambiguous	6 (8)
**Baseline PHA, *n* (%)**	​
<5,000 cpm	48 (63)
>5,000 cpm	28 (37)
**Received immune suppression, *n* (%)**	​
Yes	49 (64)
No	27 (36)

a CHARGE/*CHD7* numbers are based on phenotype or genetic testing.

### Impact of HLA matching

**Figure 2.**
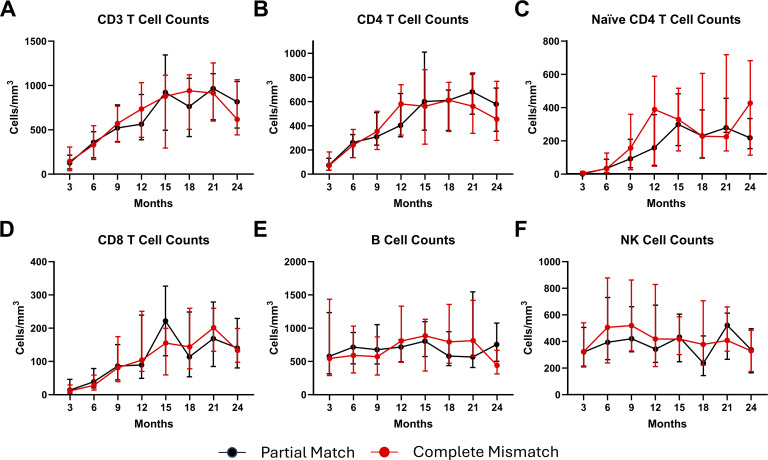
**Longitudinal comparison of median lymphocyte subset counts** **after** **CTTI among participants with complete (6/6) HLA mismatch vs. partial (>1) match. (A–F)** Median total cell counts with interquartile range for lymphocytes subsets at 3-mo intervals (y-axis) through 24 mo after CTTI (x-axis) among participants with complete HLA mismatch (6/6), shown in red, or partial HLA match (>1 allele), shown in black. Comparison by Mann–Whitney U test. No significant differences in the median CD3 (A), CD4 (B), naive CD4 (C), or CD8 (D) T cell counts. No significant differences in the median B cell (E) or NK cell (F) counts. P values for comparison of each 3-mo interval are provided in Table S1. Lymphocyte enumeration results were censored in three participants at the time they received alemtuzumab treatment at one at 3 mo and two at 12 mo, respectively.

Among the HLA subgroup (*n* = 70), there were no significant differences by 12 mo after CTTI in median CD3 (P = 0.33), CD4 (P = 0.34), naive CD4 (P = 0.64) or CD8 (P = 0.46) T cell counts, B cell counts (P = 0.18), or natural killer (NK) cell counts (P = 0.65) between participants with complete HLA mismatch and those with partial matching (Fig. 2). Median cell T cell counts and P values for comparisons at individual 3-mo periods extending from 3 to 24 mo following CTTI are provided in Table S2.

### Impact of baseline T cell function

**Figure 3.**
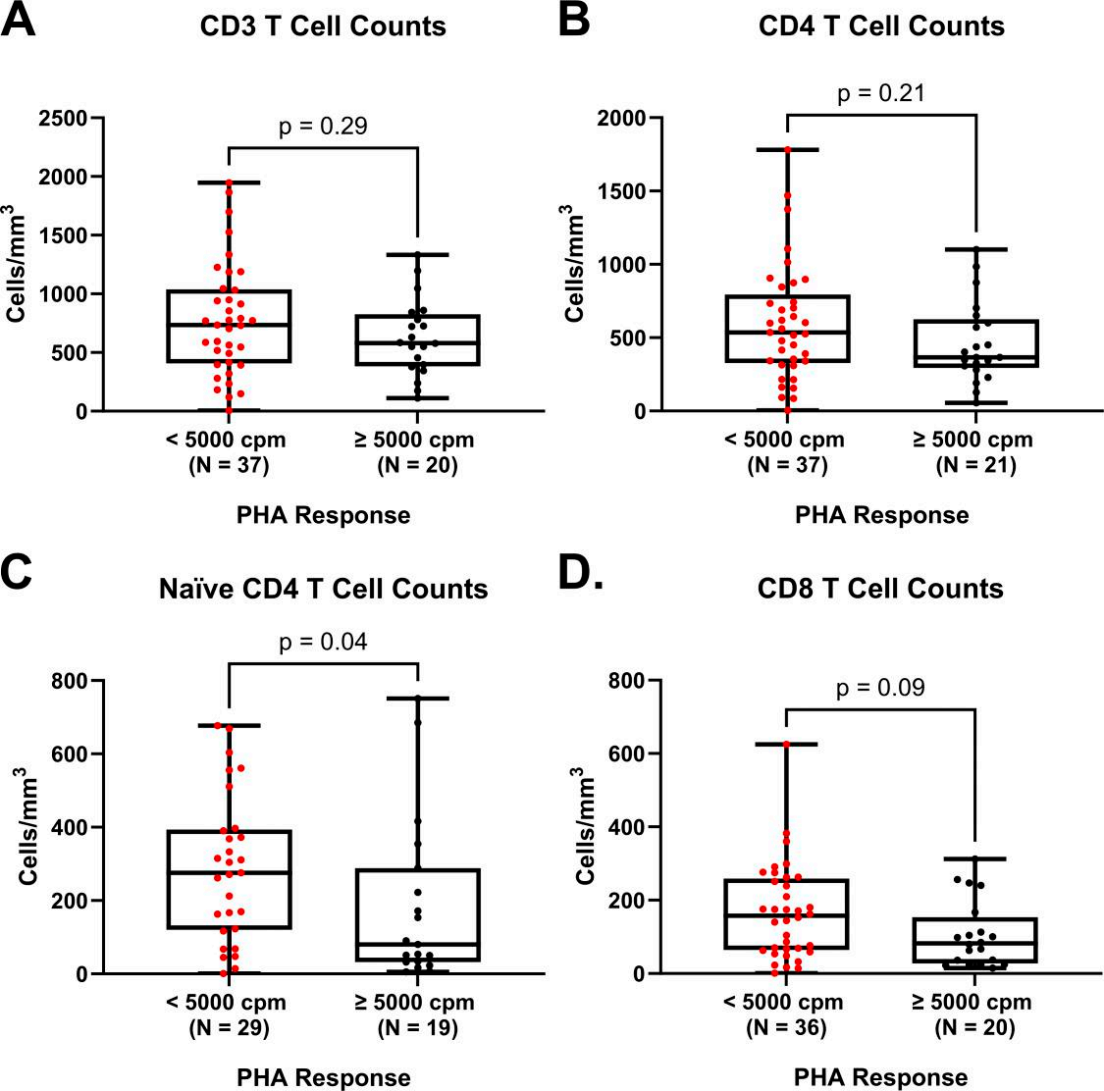
**12** **-mo post-CTTI comparison of median T cell subset counts among participants with PHA response <5,000 cpm vs. >5,000 cpm.** Median T cell subset counts at 12-mo after CTTI among participants with baseline PHA *>*5,000 cpm (black dots) versus <5,000 cpm (red dots). Comparisons by Mann–Whitney U test. No significant differences in the median CD3, CD4, or CD8 T cell counts. There is a significant difference in the median naive CD4 counts favoring baseline PHA <5,000 cmp. Flow data for one participant is excluded due to previous receipt of alemtuzumab. **(A)** Median (Q1; Q3) CD4 T cell counts (cells/mm^3^) for PHA >5,000 cpm and <5,000 cpm are 366 (294; 626) and 535 (328; 794), respectively. **(B)** Median (Q1; Q3) naive CD4 T cell counts (cells/mm^3^) for PHA >5,000 cpm and <5,000 cpm are 80 (32; 289) and 276 (120; 394), respectively. **(C)** Median (Q1; Q3) CD3 T cell counts (cells/mm^3^) for PHA >5,000 cpm and <5,000 cpm are 581 (383; 827) and 734 (407; 1,038), respectively. **(D)** Median (Q1; Q3) CD8 T cell counts (cells/mm^3^) for PHA >5,000 cpm and <5,000 cpm are 82 (28; 154) and 158 (64; 259), respectively. PHA, phytohemagglutinin.

There were no significant differences in T cell reconstitution when comparing median CD3, CD4, or CD8 T cell counts at 12 mo after CTTI between those participants with a baseline T cell function based on PHA proliferative responses of >5,000 cpm and those with responses <5,000 cpm (Fig. 3). However, the median naive CD4 T cell counts at 12 mo after CTTI were significantly higher among those participants with low baseline PHA proliferative responses <5,000 cpm (P = 0.044).

**Figure 4.**
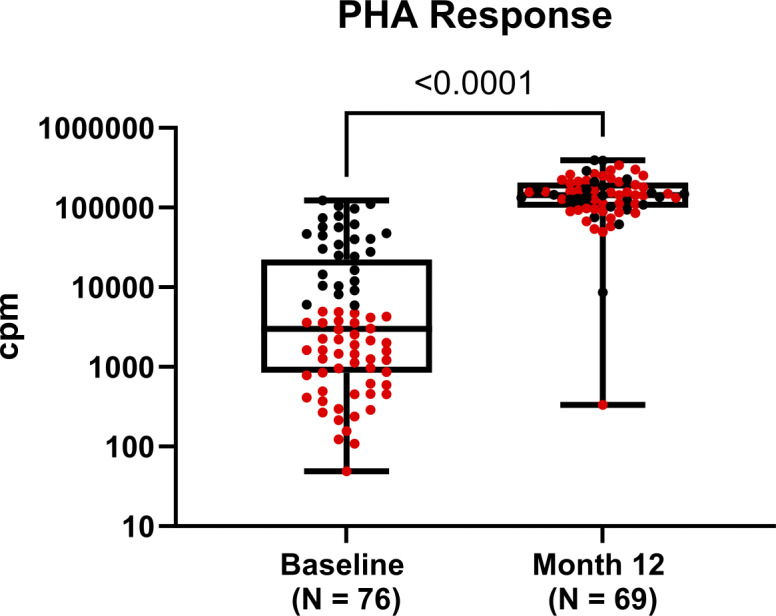
**Comparison of PHA response at baseline and 12 mo** **after** **CTTI.** Median PHA (cpm) response among participants at baseline versus 12 mo after CTTI. Comparison by Wilcoxon-matched pairs signed-rank test. There is a significant increase in median PHA response from baseline (pre-CTTI) to 12 mo after CTTI. Red dots represent participants that had PHA responses <5,000 cpm at baseline, prior to CTTI, while black dots represent participants that had PHA responses >5,000 at baseline. 12-mo PHA response data are excluded for three participants who received alemtuzumab after CTTI as treatment for autoimmunity. PHA proliferation responses were not available in four participants at 12 mo after CTTI. Median (Q1; Q3) PHA response (cpm) for baseline and 12 mo after CTTI are 2,988 (849; 22,271) and 143,485 (99,854; 206,169), respectively.

PHA proliferative responses at 12 mo included results that were closest to the 12-mo post-CTTI time point, ranging from 8.9 to 24.9 mo with a median (Q1; Q3) of 12.2 mo (11.2; 13.6). PHA responses normalized in 97% (67/69) of participants by 12 mo after CTTI regardless of the pre-CTTI proliferation results (Fig. 4). Among the two participants who did not achieve normal PHA responses, as shown in Fig. 4, one had a 12-mo post-CTTI proliferation response of 8,595 cpm, but by 2 years after CTTI, the mitogen response had normalized to 263,656 cpm (data not shown). The other participant’s 12-mo post-CTTI proliferation response was 334 cpm, with a corresponding absence of naive CD4 T cells, and did not achieve normalization by >2 years after CTTI. The participant’s clinical course following implantation was complicated by cardiac arrest within 1 mo following CTTI, along with severe autoimmune comorbidities managed with immune-suppressing agents including frequent systemic corticosteroids. The combination of these events likely contributed to insufficient reconstitution.

### Impact of immune suppression

Immune suppression was administered before or after CTTI in 49/76 (64%) of participants, either as treatment for autoimmunity or for those whose baseline T cell numbers and function indicated a high risk of allograft rejection (19). Use of immune suppression across the IND protocols was not standardized and evolved with time. The most common immune suppressive medications administered in the week prior to CTTI were rabbit antithymocyte globulin (rATG) for 44/49 (87.8%) participants, with the addition of calcineurin inhibitors in 27/49 (49%), either cyclosporin in 25/49 (51%) or tacrolimus in 2/49 (4%) (15) Other, less frequently administered therapies prior to CTTI included mycophenolate mofetil (*n* = 4), alemtuzumab (*n* = 3), horse antithymocyte globulin (*n* = 2), rituximab (*n* = 2), sirolimus (*n* = 1), daclizumab (*n* = 1), infliximab (*n* = 1), azathioprine (*n* = 1), daratumumab (*n* = 1), and basiliximab (*n* = 1). No other immune modulating medications were used during the studies.

**Figure 5.**
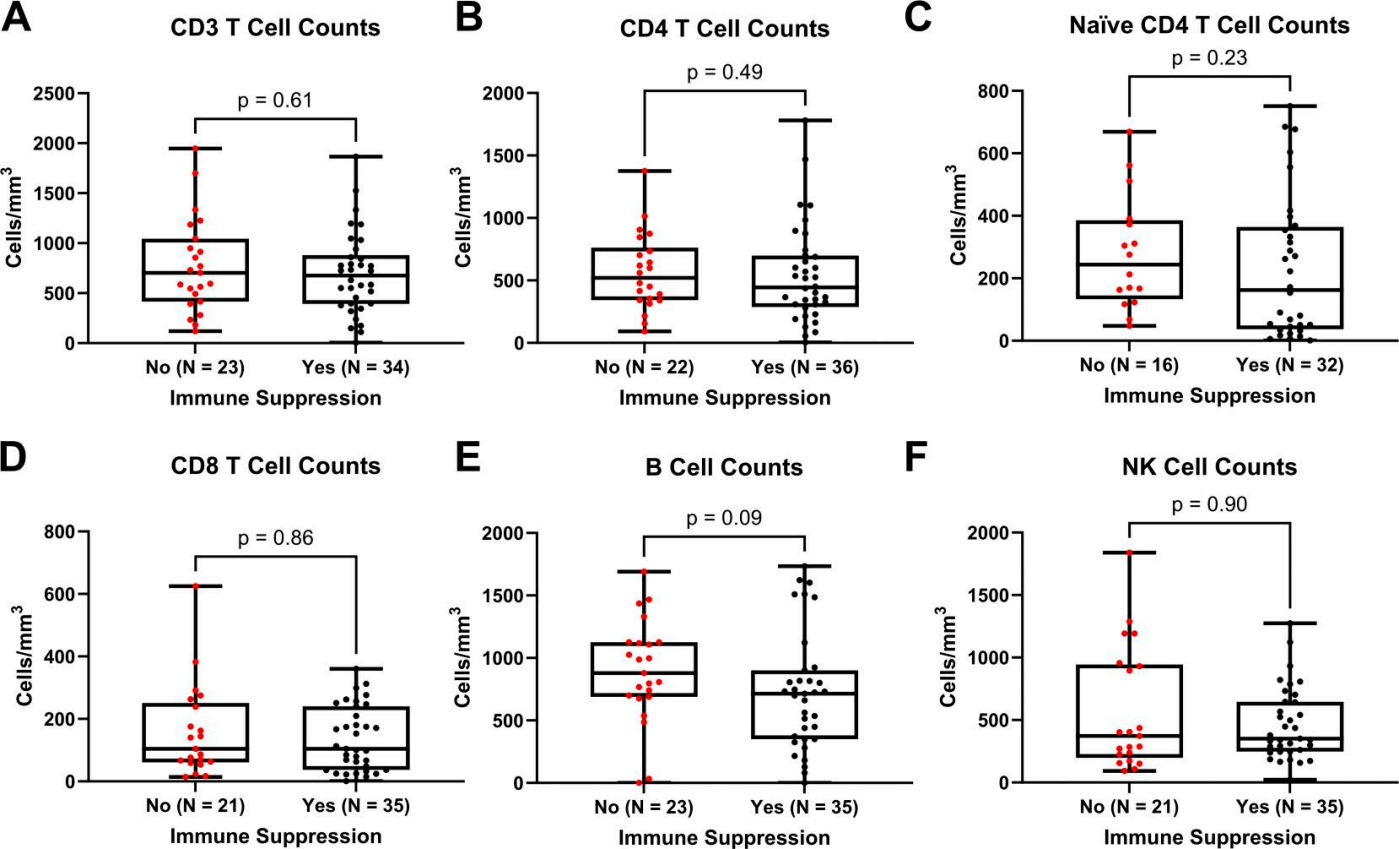
**Comparison of median lymphocyte subset counts based on immunosuppression status.** Median lymphocyte subsets at 12-mo after CTTI among participants who received any immunosuppressive therapies (black circles) versus those who did not (red circles). Comparison by Mann–Whitney U test. No significant differences in the median CD3, CD4, naive CD4, CD8, B cell, or NK cell counts. Lymphocyte enumeration results for one participant who was treated with alemtuzumab were excluded. **(A)** Median (Q1; Q3) CD4 T cell counts (cells/mm^3^) for immunosuppression “no” and “yes” are 520 (343; 762) and 444 (288; 699), respectively. **(B)** Median (Q1; Q3) naive CD4 T cell counts (cells/mm^3^) for immunosuppression “no” and “yes” are 244 (133; 386) and 163 (37; 365), respectively. **(C)** Median (Q1; Q3) CD3 T cell counts (cells/mm^3^) for immunosuppression “no” and “yes” are 703 (416; 1,045) and 676 (392; 880), respectively. **(D)** Median (Q1; Q3) CD8 T cell counts (cells/mm^3^) for immunosuppression “no” and “yes” are 104 (61; 251) and 104 (37; 240), respectively. **(E)** Median (Q1; Q3) B cell counts (cells/mm^3^) for immunosuppression “no” and “yes” are 879 (689; 1,126) and 714 (351; 900), respectively. **(F)** Median (Q1; Q3) NK cell counts (cells/mm^3^) for immunosuppression “no” and “yes” are 373 (198; 944) and 351 (248; 646), respectively.

At 12 mo after CTTI, there were no significant differences in the median CD3 (P = 0.61), CD4 (P = 0.49), naive CD4 (P = 0.23), or CD8 (P = 0.86) T cell counts, B cell counts (P = 0.09), or NK cell counts (P = 0.90) between participants who received immune suppression either before, during, or after CTTI compared to those who did not (Fig. 5).

### Impact of age at time of implantation

**Figure 6.**
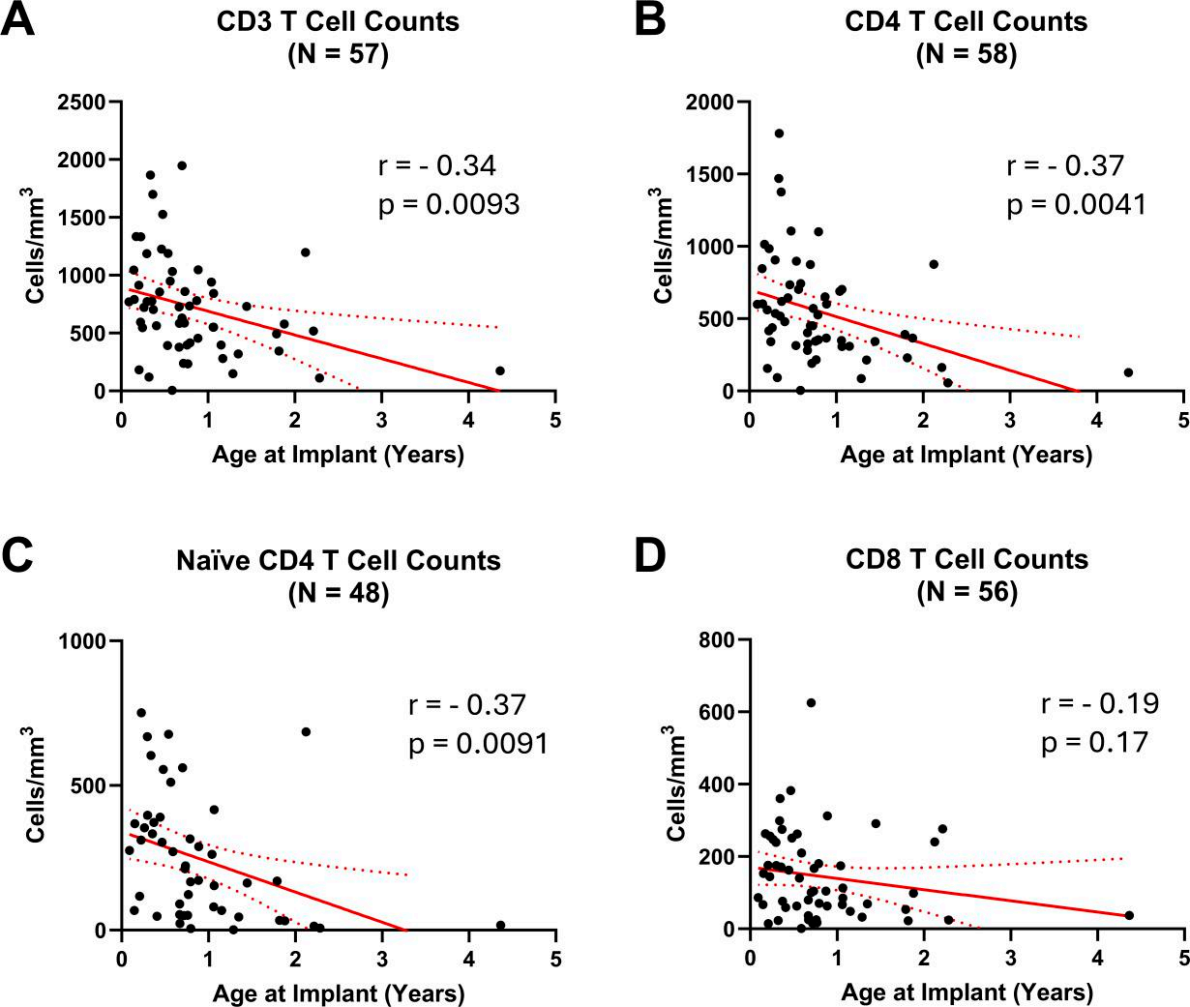
**Correlation between median T cell subset counts at 12 mo** **after** **CTTI and participant age (years) at the time of thymus implantation.** Pearson correlation between age (years) and median CD3, CD4, naive CD4, and CD8 T cell counts. Lymphocyte enumeration results for the participant who received alemtuzumab at 3 mo after CTTI are excluded. **(A)** Median (Q1; Q3) CD4 T cell count (cells/mm^3^) is 465 (313; 710). **(B)** Median (Q1; Q3) naive CD4 T cell count (cells/mm^3^) is 192 (52; 371). **(C)** Median (Q1; Q3) CD3 T cell count (cells/mm^3^) is 703 (397; 945). **(D)** Median (Q1; Q3) CD8 T cell count (cells/mm^3^) is 104 (55; 240).

Across the entire cohort (*n* = 76), the median (Q1; Q3) age at time of implantation was 8.9 mo (4.6; 14.1). Older age at implantation had a weak, but significant, negative correlation with absolute CD3, CD4, and naive CD4 T cell counts at 12 mo after CTTI (Fig. 6). There was not a significant correlation between age at implantation and absolute CD8 cell counts at 12 mo after CTTI.

### Multivariable logistic regression analysis for reconstitution after CTTI

Multivariable logistic regression analysis was undertaken to assess the effect of the immune suppression, baseline T cell mitogen proliferative responses, age at implantation, and genetic/syndrome etiology on naive T cell reconstitution at 12 mo after CTTI, defined as a naive CD4 T cell count >100 cells/mm^3^. By 24 mo after CTTI, 79% of the participants with available data achieved this milestone. The analysis included 44 participants, as the six participants with ambiguous HLA typing, 25 participants who did not have available 12-mo naive T cell results, and one participant who received alemtuzumab prior to 12 mo after CTTI were all excluded. Table 3 shows the results of the multivariable logistic regression. There was no significant association in the odds of naive T cell reconstitution based on having received immune suppression, baseline PHA >5,000 cpm, or genetic/syndromic etiology. However, younger age at time of implantation was a significant predictor, with the odds of reconstitution at 12 mo after implantation decreasing by 11% for every 30-day increase in age at time of implantation.

**Table 3.** Multivariable logistic regression

Variable	OR	CI	P value
Immune suppression, yes	0.39	0.04–2.96	0.38
Baseline PHA >5,000	0.42	0.07–2.19	0.31
Age at implantation (30 days)	0.89	0.79–0.98	**0.03**
**Genetic/syndromic etiology**	​	​	​
22q-related variant	**Reference**	​	​
IDM	2.72	0.50–19.5	0.27
No etiology identified	5.09	0.36–163.5	0.28

Multivariable logistic regression, including variables of interested selected through stepwise backward selection. Bold indicates significant value. IDM, infant of diabetic mother*.* OR, odds ratio; CI, confidence interval.

## Discussion

Most children who receive CTTI demonstrate absolute T cell numbers and percentages of recent thymic emigrants below the age adjusted levels seen in healthy children (20, 21). However, the majority of recipients express a diverse T cell repertoire, display normal T cell function, and can effectively wean infection prophylaxis (21, 22, 23). Many factors potentially impact thymus function and the extent of T cell immune reconstitution following implantation (20). Infection preceding or following implantation, preexisting autoimmunity, donor or recipient age, and use of immune suppressive medications have all been implicated in affecting the magnitude of T cell reconstitution (16, 24). Serious infections occurred after CTTI in over 50% of the cohort and new viral infections in 75%. However, testing for viral and other infections was inconsistent after CTTI, impairing the analysis of the relationship between infection and immune reconstitution. This study focuses on specific factors that may also influence T cell outcomes following CTTI: the extent of HLA mismatch, T cell function prior to CTTI (as measured by mitogen proliferation), receipt of immune suppressing agents, and recipient age.

RETHYMIC prescribing information only requires that recipients be screened for anti-HLA antibodies prior to receiving treatment (15). Recipients who are positive for anti-HLA antibodies need to receive RETHYMIC from a donor who does not express the sensitized HLA alleles to prevent allograft rejection (21, 25, 26, 27). However, HLA matching of the donor thymus to the recipient is not required. In the context of solid organ transplantation, such as renal transplant, partial HLA matching can induce tolerance and improve long-term graft survival when compared to complete mismatch. However, these studies required thousands of participants to show the effect. Our study demonstrates that children with CA can achieve immune reconstitution of total and naive T cells regardless of the degree of HLA mismatch between thymus donor and recipient. It is possible that with time and a larger number of children being treated with thymus implantation, an association may be identified that supports partial HLA matching to improve self-tolerance and enhanced T cell reconstitution. However, fewer than 200 children have received CTTI in the United States, making this outcome challenging to evaluate in context of other potentially influential variables (16). Similarly, limited donor tissue availability makes complete HLA matching between donor and recipient impractical.

**Figure 7.**
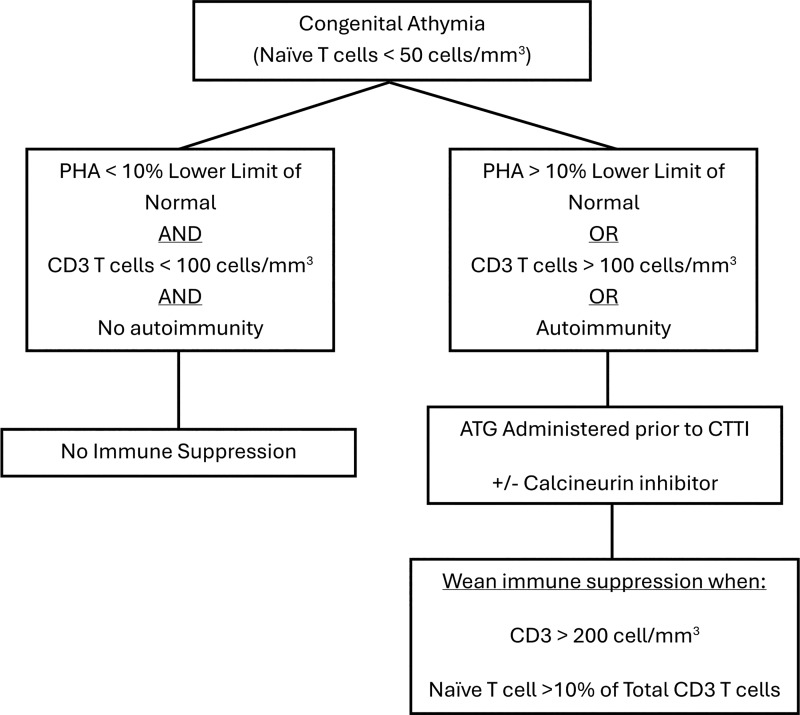
**Immune suppression protocol.** Proposed decision algorithm for determining use of immune suppression in infants with CA and criteria for weaning immune suppression after implantation. ATG, antithymocyte globulin.

As can be seen in patients with typical SCID in the early years after hematopoietic stem cell transplant, successful T cell reconstitution can be achieved in patients with CA without the need for pretransplant conditioning (14, 28, 29). In our study, participants with CA who had no circulating T cells, minimal T cell function, and no evidence of autoimmunity or host autoreactive T cells representing oligoclonal T cell expansion were, in many cases, not treated with immune suppression before or after implantation (19). It was presumed that the lack of T cell function would prevent allograft rejection of the donor thymus stroma cells (30). Gradual infiltration of recipient antigen-presenting cells into the allograft would eventually lead to the induction of self-tolerance, preserve graft function, and lead to normal T cell development (27). In the IND trials, these participants were referred to as a typical phenotype as opposed to those participants with functional T cells or autoimmunity, referred to as an atypical phenotype (16). In contrast, participants with CA and evidence of T cell function require immune suppression to prevent allograft rejection and curtail autoimmunity (19). In the analysis for this study, we compared T cell reconstitution among participants with CA who exhibited baseline mitogen proliferation responses <5,000 cpm (<10% of the lower limit of normal for the laboratory) (31) to those with mitogen proliferation responses ≥5,000 cpm. While there were no significant differences in total T cell numbers and T cell function 1 year after CTTI, total naive T cells were significantly higher in the cohort with baseline proliferation responses <5,000 cpm. These results suggest that optimal restoration of thymus function occurs when implantation is performed in the setting of minimal baseline T cell function and that thymopoiesis and immune reconstitution may be hindered by the presence of host autoreactive oligoclonal T cells and ongoing autoimmunity. Unlike some forms of SCID, patients with CA do not have an intrinsic defect in T cell function. Low mitogen proliferation responses more likely reflect very low T cell numbers as has been shown when comparing T cell proliferation using tritiated thymidine uptake compared to flow cytometry–based T cell functional assay (31). In the current management of CA, immune suppression is administered if the T cell functional assay is >10% of the lower limit of normal, regardless of the assay type (Fig. 7).

The majority of CTTI recipients included in our study received immune suppression, even those with baseline mitogen proliferation responses <5,000 cpm. This primarily occurred due to the development of autoimmunity. 53 participants had features of autoimmunity (e.g., rash/dermatitis, lymphadenopathy, cytopenia(s), alopecia, hepatitis, and hypothyroidism) prior to CTTI. Of those, 37 had notable T cell infiltration on skin biopsy and oligoclonal T cell expansion, as previously described (3, 18, 19, 21, 30, 32). Over the years of the IND studies, which spanned from 1997 to 2021, the therapeutic approach for the use of immune suppression evolved, as reflected in the broad array of immune suppressive medications used in the protocols. As early as 2008, it was evident that administration of immune suppression did not appear to adversely affect T cell reconstitution following CTTI (24). Additionally, post-CTTI thymus allograft biopsies have shown robust thymopoiesis among participants with the atypical phenotype who received postimplantation immune suppression (20, 30). In our study, three participants received alemtuzumab after CTTI for management of autoimmunity due to host autoreactive oligoclonal T cells, which has been shown to be effective with similar disease states such as GVHD (33, 34). Analysis of our larger cohort demonstrated that immune suppression administration consisting of rATG or calcineurin inhibitors, pre- and/or after CTTI—whether for the treatment of humoral-mediated autoimmunity or presence of host autoreactive T cells or for the prevention of allograft rejection—did not impair immune reconstitution. However, systemic corticosteroids have been implicated in damaging thymus tissue grafts (30).

Younger age at time of CTTI appears to play a key role in optimal T cell reconstitution following implantation. When examined independently, there is a correlation between implantation at a younger age with improved CD4 and naive CD4 T cell reconstitution by 12 mo after implantation. This finding is further supported by a multivariable analysis whereby each 30-day increase in age without receiving a thymus implantation was associated with an 11% decrease in the odds of naive T cell reconstitution at 12 mo after implantation. However, the smaller cohort size limited the number of variables, which could be included within the regression modeling to avoid overfitting the data set. Thus, the results primarily reflect the cohort and become less generalizable and should be interpreted cautiously. Studies of rare diseases are often hampered by small sample size, limiting the number of variables that could be reasonably included within regression modeling. Demographic variables such as race and ethnicity were not examined due to the ambiguity in reporting of these variables, and ascertaining race and ethnicity information from the electronic medical record has been demonstrated to be unreliable (35, 36, 37). However, as in the case of infants with SCID, older age related to delay of CTTI increases the risk of infection, development of autoimmunity, and oligoclonal T cell expansion likely increasing morbidity and mortality (20, 21, 24). These factors will need to be examined collectively to determine the optimal timing for CTTI. To date, detailed analysis of T cell reconstitution following CTTI has primarily focused on total T cell numbers and clinical outcomes, including survival, the capacity to clear preexisting infections, and wean prophylaxis (16, 20). Notably, a substantial proportion of participants, ∼40%, did not have naive T cells >100 cells/mm^3^ at 12 mo, and most children had total T cell counts below the 10^th^ percentile for age yet do well clinically (16). Clearly better biomarkers are needed to fully assess reconstitution of cellular immunity following CTTI.

The degree of HLA matching does not appear to impact T cell reconstitution, so currently, implantation is carried out using mismatched donors except when the recipient exhibits a positive panel reactive antibody result (15). Common criteria for thymus implantation includes naive T cells <50 cells/mm^3^, a known genetic defect or embryopathy that affects thymus development, and no genetic evidence of SCID. Infants who have absent or very low T cell numbers and function (as measured by mitogen response) with no evidence of autoimmunity can reconstitute T cell immunity following CTTI without the need for immune suppression. Evidence of T cell function or autoimmunity prior to implantation will require immune suppression. Current immune suppression protocols include rATG for three consecutive days prior to implantation with the addition of a calcineurin inhibitor (Fig. 7) (15, 16). Immune suppression can be weaned when there is evidence of thymus output, generally a minimum of 6 mo after CTTI when CD3 T cells are >200 cells/mm^3^ with at least 10% being naive T cells, and no evidence of host autoreactive T cells or autoimmunity (38). The younger the age at time of implantation, the better the degree of naive T cell reconstitution. Early diagnosis and prompt referral for CTTI are therefore critical determinants of successful thymic reconstitution.

## Materials and methods

### Study design

This study involved retrospective analysis of previously collected data for participants enrolled in IND 9836, consisting of 10 prospective, single-arm, open-label protocols initiated in 1993 and completed in 2023 approved by the Duke Health Institutional Review Board (Pro00104164; Umbrella Protocol for Thymus Transplantation Protocols). Participants were children under the age of 5 years with written consent obtained from parent(s) or guardian(s). The database is maintained by Sumitomo Pharma America, Inc., who provided study data that was de-identified and coded prior to analysis. Eligibility criteria included having a clinical phenotype/molecular variant associated with CA, T cell counts <50 cells/mm^3^ or naive T cell (CD3/CD4/CD45RA/CD62L) counts <50 cells/mm^3^ on two separate occasions, and absence of genetic defects associated with SCID (39). Syndromic features associated with CA included congenital heart defects in 89.7% (*n* = 87) and hypoparathyroidism, defined as low serum calcium levels, in 84.5% (*n* = 82). Exclusion criteria included heart surgery within 4 wk prior to CTTI or anticipated within 3 mo after CTTI, poor surgical candidate, HIV infection, prior attempts at immune reconstitution (e.g., hematopoietic stem cell transplant or previous thymus implant, etc.), ventilator dependence, and cytomegalovirus infection for patients requiring immune suppression. Assessments for autoimmunity were based on medical history extracted from the database including skin biopsy of rashes, report of autoimmune thrombocytopenia, neutropenia or anemia, adenopathy, enteropathy, or autoimmune endocrinopathy.

The primary outcomes evaluated were absolute CD3, CD4, CD8, and naive CD4 (CD3/CD4/CD45RA/CD62L) T cell counts at regular intervals between 3 and 24 mo after CTTI. Secondary outcomes evaluated were absolute B cell and NK cell counts at regular intervals between 3 and 24 mo after CTTI and T cell proliferation response to PHA up to 24 mo after CTTI. Many of the participants returned to their referring centers after CTTI, which resulted in some variation in the sequence of the postimplantation lymphocyte enumeration, thus the number of participants who had flow cytometry performed at each time interval after CTTI varied. Three participants received alemtuzumab for autoimmune comorbidities after CTTI, and their lymphocyte enumeration and PHA response results following receipt of alemtuzumab were excluded from analysis.

### Thymus donor tissue

Cultured thymic tissue is manufactured using tissue obtained from unrelated, healthy donor infants age ≤9 mo who are undergoing elective cardiac surgery for congenital heart disease. The thymic tissue is routinely excised to allow the surgeon access to the heart (15, 16). Parent(s) of the donor provide consent to use their child’s thymus tissue (21). Donor infants and their mothers are screened for the presence of pathogens, and infants are tested to assure they have normal cellular immunity (21).

Donated thymus tissue manufacturing involves 12–21 days in culture to produce the tissue used for implantation (15, 17, 18). The manufacturing process removes most of the donor T cells from the thymus tissue, but epithelial viability and tissue structure are preserved. The product is thoroughly tested for sterility, endotoxin, and *Mycoplasma* spp. before release for administration (15).

### HLA typing of thymus donors and recipients

HLA typing was performed in the Duke University Hospital Clinical Transplant Immunology Laboratory using high-resolution molecular-based sequencing (40). Participants who had low-resolution sequencing resulting in ambiguity with respect to the exact amino acid sequence were excluded from the HLA analysis. The degree of HLA mismatch at A, B, and DRB1 alleles was classified as either complete mismatch (mismatch of all six alleles) or partial match (match of at least one allele). Per protocol, all patients were screened for panel reactive antibodies prior to CTTI (16). If positive, a donor was selected who did not express the impacted allele.

### Lymphocyte function measured by proliferation response to PHA mitogen

T cell function prior to and following CTTI was assessed using proliferation responses to PHA performed 1 mo prior to CTTI and up to 24 mo after CTTI. Assays were performed in the Duke University Health System Clinical Immunology Laboratory. Briefly, peripheral blood mononuclear cells were isolated using density gradient centrifugation and SepMate tubes and incubated at 37°C, 5% CO_2,_ and 100% humidity for 3 days in the presence of three different concentrations of PHA. Culture was performed in triplicate in 96-well microtiter plates pulsed with 1 µCi ^3^H thymidine for 6 h and then harvested with a Revvity automated sample harvester. Thymidine uptake was measured using a PerkinElmer MicroBeta^2^ Counter and optimal uptake recorded in cpm. Participants with baseline T cell proliferative response to PHA <5,000 cpm were classified as having no T cell functional capacity, whereas those with PHA responses >5,000 cpm were classified as having measurable T cell function (31).

### Immune suppression treatment for autoimmunity or to prevent CTTI rejection

According to protocols, the extent of immune suppression was based on the baseline PHA proliferation response (16). Participants with baseline T cell proliferative response to PHA <5,000 cpm, total T cells <100 cells/mm^3^, and no evidence of autoimmunity did not receive immune suppression. Those with a baseline T cell proliferative response to PHA >5,000 cpm, CD4 T cells >100 cells/mm^3^, or autoimmunity received immune suppression, most commonly 3 days of rATG, in combination with low-dose glucocorticoids to prevent adverse reactions and a calcineurin inhibitor following implantation (16, 19). Some participants received additional immune suppression with glucocorticoids, mycophenolate mofetil, alemtuzumab, horse antithymocyte globulin, rituximab, sirolimus, daclizumab, infliximab, azathioprine, daratumumab, and basiliximab. For the analysis of outcomes, participants were grouped based on having received any immune suppression therapy either before, during, or after CTTI.

### Statistical analysis

Univariable statistical analyses were performed using Version 9.5.1 or later of GraphPad Prism. Data were subjected to Shapiro–Wilk normality tests prior to analysis. Descriptive statistics were used to summarize participant characteristics, including sex, race, ethnicity, genetic/syndromic etiology, age at time of CTTI, baseline T cell proliferation response to PHA, and whether a participant received immune suppression. Differences in lymphocyte subsets between participants with complete HLA mismatch versus partial HLA match, participants who received immune suppression versus those who did not, and participants with baseline T cell proliferation response to PHA <5,000 cpm versus ≥5,000 cpm were determined by Mann–Whitney U tests. P values <0.05 were considered statistically significant. Pearson correlation was used to evaluate the correlation between T cell subsets and age at time of implantation.

Multivariable logistic regression analysis to assess the outcome of naive T cell reconstitution after CTTI was performed using R (v4.5.0; R Core Team 2025 and RStudio) (v2025.05.0+496; Posit Team, 2025). Participants were classified as having achieved reconstitution at 12 mo after CTTI if their naive CD4 T cell count was >100 cells/mm^3^. A single participant who received alemtuzumab prior to 12 mo was excluded from the analysis. Independent variables included participant sex, genetic/syndromic etiology, HLA mismatch, baseline PHA, age at time of implantation, and immune suppression. Race and ethnicity data were excluded as the reported race and ethnicity were ambiguous for many participants in the database, and collection of this information through the electronic medical record is unreliable (35, 36, 37). Underlying genetic/syndromic etiology categories were defined as: 22q-related variant (having 22q11.2DS, phenotypic characteristics of CHARGE syndrome and/or identified CHD7 mutation, PAX1 deficiency, or FOXN1 deficiency), infant of a diabetic mother, or no etiology identified. Stepwise backward selection was employed to remove independent variables from the model with a P value >0.5.

### Online supplemental material

Table S1 shows alemtuzumab administration after CTTI. Table S2 shows 3-mo median cell count analysis after CTTI.

## Supplementary Material

Table S1 shows alemtuzumab administration after CTTI.

Table S2 shows 3-mo median cell count analysis after CTTI.
