# Supplementary material for: Rebooting immunity in congenital athymia: Factors impacting reconstitution with thymus implantation
Source: J Hum Immun. 2026 May 26;2(4):e20260016. doi: 10.70962/jhi.20260016 (PMC13205136; doi:10.70962/jhi.20260016)
Supplement: Table S1 — shows alemtuzumab administration after CTTI. [file jhi_20260016_tables1.docx]

**Supplemental material**

**Supplemental Table 1: Alemtuzumab Administration Post-CTTI**

|  | Month 3 | Month 6 | Month 9 | Month 12 | Month 15 | Month 18-21 | Post-Month 24 |
| --- | --- | --- | --- | --- | --- | --- | --- |
|  | **(Cells/mm^3^)** | | | | | | |
| **Participant 1** |  |  |  |  |  |  |  |
| Vital status |  |  |  |  |  |  | Alive |
| CD4 | 76 | 158.18 | 310.44 | 1100 |  | 55 | 232 |
| Naïve CD4 | 0 |  | 6 | 5.5 |  |  |  |
| CD3 | 1259 | 458.91 | 680.81 |  |  | 137 | 348 |
| CD8 | 1165 | 295.21 | 377.4 |  |  | 83 | 105 |
|  |  |  |  |  |  |  |  |
| B | 1 | 54.37 | 72.12 |  |  | 5 | 557 |
| NK | 137 | 269.25 | 298.68 |  |  | 162 | 308 |
| PHA |  |  |  | ND |  |  | 5,822^†^* |
| **Participant 2** |  |  |  |  |  |  |  |
| CD4 | 70 | 320 | 134 | 85 | 0 | 10 | 354 |
| Naïve CD4 | 0 | 3 | 5 | 1 | 0 | 0 |  |
| CD3 | 82 | 349 | 163 | 148 | 0 | 17 | 433 |
| CD8 | 5 | 22 | 21 | 32 | 0 | 2 | 75 |
| B | 137 | 306 | 246 | 128 | 0 | 2 | 34 |
| NK | 212 | 144 | 204 | 807 | 397 | 262 | 254 |
| PHA |  |  |  | ND |  | 4523 | 19,202^†^* |
| **Participant 3** |  |  |  |  |  |  |  |
| CD4 | 292 | 66 | 121 | 140 | 123 | 190 | 519 |
| Naïve CD4 | 2 | 1 | 1 |  | 4 | 28 | 98 |
| CD3 | 390 | 160 | 184 | 207 | 167 | 254 | 697 |
| CD8 | 62 | 75 | 45 | 61 | 39 | 50 | 141 |
| B | 150 | 151 | 47 | 104 | 75 | 121 | 388 |
| NK | 123 | 61 | 111 | 340 |  | 227 | 748 |
| PHA** |  |  |  | ND |  |  | 83.5^†^** |
| Vital Status: All participants are currently alive at last communication. Range of follow-up post-CTTI (2 – 7.5 years) | | | | | | | |

Numbers in black and red denote lymphocyte enumeration (cells/mm^3^), pre- and post-Alemtuzumab administration, respectively. ^†^PHA proliferation assays were performed by either measuring ^3^H-thymidine cellular uptake to measure proliferation *(counts per minute) or by flow cytometric gating of the CD3^+^ T-cell population, with results reported as percentage of CD3^+^ T-cells undergoing proliferation, **(% of CD3).
