# Supplementary material for: Rebooting immunity in congenital athymia: Factors impacting reconstitution with thymus implantation
Source: J Hum Immun. 2026 May 26;2(4):e20260016. doi: 10.70962/jhi.20260016 (PMC13205136; doi:10.70962/jhi.20260016)
Supplement: Table S2 — shows 3-mo median cell count analysis after CTTI. [file jhi_20260016_tables2.docx]

**Supplemental material**

**Supplemental Table 2: 3-Month Median Cells Count Analysis Post-CTTI**

|  | Month 3 | Month 6 | Month 9 | Month 12 | Month 15 | Month 18 | Month 21 | Month 24 |
| --- | --- | --- | --- | --- | --- | --- | --- | --- |
| **Naïve CD4 T Cells** |  |  |  |  |  |  |  |  |
| Partial Match* | 1.05 | 34.14 | 92.00 | 158.3 | 299.1 | 230.1 | 278.9 | 218.5 |
| Complete Mismatch* | 2.28 | 23.0 | 116.3 | 289.8 | 244.9 | 169.1 | 166.9 | 318.9 |
| p-value | 0.88 | 0.78 | 0.75 | 0.64 | 0.63 | 0.86 | 0.54 | 0.69 |
| **CD4 T Cells** |  |  |  |  |  |  |  |  |
| Partial Match* | 71.50 | 261.0 | 308.0 | 402.7 | 600.0 | 610.0 | 681.0 | 579.0 |
| Complete Mismatch* | 73.00 | 241.0 | 353.0 | 580.3 | 561.0 | 612.3 | 561.0 | 455.0 |
| p-value | 0.72 | 0.94 | 0.80 | 0.34 | 0.43 | 0.73 | 0.48 | 0.42 |
| **CD3 T Cells** |  |  |  |  |  |  |  |  |
| Partial Match* | 126.0 | 358.5 | 521.0 | 563.0 | 920.0 | 762.0 | 965.0 | 815.0 |
| Complete Mismatch* | 144.0 | 331.0 | 572.0 | 734.0 | 878.0 | 940.5 | 914.5 | 618.0 |
| p-value | 0.94 | 0.77 | 0.97 | 0.33 | 0.23 | 0.42 | 0.90 | 0.52 |
| **CD8 T Cells** |  |  |  |  |  |  |  |  |
| Partial Match* | 14.00 | 39.00 | 86.50 | 89.79 | 222.0 | 114.0 | 169.0 | 140.9 |
| Complete Mismatch* | 11.40 | 28.00 | 81.00 | 104.0 | 155.3 | 143.8 | 201.5 | 133.0 |
| p-value | 0.46 | 0.21 | 0.82 | 0.45 | 0.09 | 0.32 | 0.75 | 0.88 |
| **B Cells** |  |  |  |  |  |  |  |  |
| Partial Match* | 578.5 | 717.0 | 679.0 | 719.0 | 807.7 | 582.5 | 568.9 | 757.1 |
| Complete Mismatch* | 544.8 | 591.0 | 572.0 | 807.3 | 886.0 | 794.2 | 812.0 | 440.2 |
| p-value | 0.93 | 0.56 | 0.32 | 0.18 | 0.68 | 0.31 | 0.90 | 0.06 |
| **NK Cells** |  |  |  |  |  |  |  |  |
| Partial Match* | 320.0 | 393.5 | 420.0 | 342.0 | 432.0 | 236.5 | 521.0 | 337.3 |
| Complete Mismatch* | 323.0 | 506.0 | 519.9 | 419.0 | 418.0 | 377.5 | 408.0 | 330.0 |
| p-value | 0.97 | 0.72 | 0.26 | 0.65 | 0.90 | 0.17 | 0.97 | 0.98 |

*Values are shown as medians and p-values for 3-month intervals comparing median cell counts following CTTI between participants with complete HLA mismatch of all six alleles and those with partial match of at least one allele by Mann-Whitney U test. *CTTI: cultured thymic tissue implantation*
